# List of Diseases in November, in the Practice of Dr. Fothergill

**Published:** 1813-12

**Authors:** 


*List of Diseases in November, in the Practice of* Dr. Fothergill.

- Tussis et Dyspnœa ·· 34

- Catarrhus ········· 11

- Pertussis ········· 5

- Asthma ············ 2

- Peripneumonia ······ 1

- Phthisis Pulmonalis ·· 4

- Scrofula ·········· 1

- Marasmus ·········· 2

- Asthenia ·········· 5

- Hypochondriasis ···· 1

- Hysteria ·········· 2

- Hæmorrhoides ······ 2

- Morbi Infantiles ···· 6

- Typhus ············ 2

- Rheumatismus ······ 11

- Cephalalgia ········ 3

- Carditis ·········· 1

- Anasarca ·········· 1

- Vomitus ··········· 2

- Dysphagia ········· 1

- Icterus ··········· 2

- Dyspepsia ········· 5

- Gastrodynia ······· 3

- Enterodynia ······· 1

- Colica Pictonum ···· 1

- Vermes ··········· 3

MONTHLY

